# Editorial: Swarm neuro-robots with the bio-inspired environmental perception

**DOI:** 10.3389/fnbot.2024.1386178

**Published:** 2024-03-05

**Authors:** Cheng Hu, Farshad Arvin, Nicola Bellotto, Shigang Yue, Haiyang Li

**Affiliations:** ^1^School of Mechanical and Electrical Engineering, Guangzhou University, Guangzhou, China; ^2^Department of Computer Science, Durham University, Durham, United Kingdom; ^3^Department of Information Engineering, University of Padua, Padua, Italy; ^4^School of Computing and Mathematical Sciences, University of Leicester, Leicester, United Kingdom; ^5^School of Mathematics and Information Science, Guangzhou University, Guangzhou, China

**Keywords:** environmental perception, neurorobotic, bio-inspired, swarm, real-world deployment

From disaster zone exploration to environmental monitoring, robots capable of navigating complex and unpredictable environments are in high demand. Inspired by the efficiency of insect swarms, the field of neuro-robotics has seen breakthroughs in efficient environmental perception and interaction. This Research Topic, titled “*Swarm neuro-robots with bio-inspired environmental perception*,” presents a cutting-edge exploration of insect-inspired neural structures and mechanisms in neuro-robots.

The Research Topic brings together six pioneering papers, each contributing unique insights into the development and application of neuro-robots with biologically inspired perception systems. From the innovative “Mobip” model that leverages MobileNet for driving perception, to the intricate study of neural feedback in motion detection, each paper highlights the exceptional adaptability and effectiveness of neuro-robots in research domains such as swarm robotics, bionic robotics, and unmanned systems. The overarching aim of this Research Topic is not only to showcase the advancements in neuro-robotic technologies but also to deepen our understanding of how these biologically inspired systems can revolutionize practical applications in fields like search and rescue, smart transportation, and beyond.

Parmar and Patton propose a novel study aiming at investigating the process of iterative learning during the reaching tasks. Their experiments reveal how our neuro-motor system recalibrates movements based on visual errors, favoring a simple, first-order model with a constant learning rate. The forgetting effects (error increasing) are observed in the unpracticed movement directions with learning effects from generalization from the practiced movement direction. This work provides a fresh approach to training in randomized movement sequences, supported by model analysis. This has notable implications for enhancing skill retention and adaptability in fields like sports coaching, neurorehabilitation, and human-machine interactions. This work is instrumental in guiding the development of more efficient and adaptable neuro-robotic systems.

Ling et al. take a deep dive into the world of insect vision, specifically how they spot tiny targets against chaotic backgrounds. Inspired by this, they construct a computational model: small target motion detection (STMD) using neural feedback. The authors employ Schauder's fixed point theorem and the contraction mapping theorem to analyze the feedback constant, as well as the existence and uniqueness of solutions within the nonlinear dynamical systems created by their feedback loop. Using a novel time-delay feedback STMD model, the study offers a robust analytical framework for the feedback mechanism found in STMD-based neural circuits. This work is pivotal in bridging biological insights with neural and robotic studies.

Ayali and Kaminka propose an innovative approach to hybrid bio-robotic research, addressing collective behavior across robotics and biology. They pinpoint a crucial gap in studying controlled collective motion using both real insects and robots. In response, the authors unveil the Nymbot-Locust bio-hybrid swarm—a pioneering platform where live locusts and custom-designed “Nymbot” robots interact in a lab setting. This allows for controlled studies of both natural and artificial swarm dynamics. By examining biological and synthetic agents simultaneously, the research promises vital insights into complex swarm interactions, showcasing the immense potential of bio-robotic collaborations in understanding natural phenomena.

Mikami et al. take inspiration from the remarkable adaptability of tubificine worm blobs to develop a simple yet effective agent-based model. Through careful observation of real worms (aggregation, responses to stimuli, movement in confined spaces), they capture the worms' ability to deform, twist, and move as a collective. In their model, each worm is represented as a flexible cross-shaped agent, and interactions with the environment are a key part of the simulation. This approach reveals the surprising ways a worm blob stretches, coils, and exploits obstacles to navigate tight spaces. This research paves the way for soft, adaptable robotic systems and offers unique insights into how swarm intelligence navigates the complexities of real-world environments.

Ye and Zhang address the need for efficient visual perception in self-driving cars with their lightweight “Mobip” network. This model simultaneously handles object detection, drivable area segmentation, and lane line detection tasks. It features a streamlined architecture: MobileNetV2 is used for shared feature extraction, followed by specialized decoders for detection and segmentation. Tested on the Berkeley Deep Drive dataset, it demonstrates a notable inference speed of 58 FPS on NVIDIA Tesla V100 hardware while maintaining accuracy across all three tasks. Ablation studies further support the effectiveness of Mobip's multi-part design. This research represents a substantial step forward in the field of autonomous vehicle technology, demonstrating how multi-task networks can be optimized for both speed and accuracy, crucial for real-world applications.

Wu et al. take a strategic approach to refining bio-inspired robotic vision. Inspired by the locust's Lobula Giant Movement Detector (LGMD) neural system, they methodically analyze existing LGMD models to distill common meta-properties essential for effective collision detection. From this analysis, they introduce the “Strategic Prototype”—a modular framework designed to guide the development of more advanced and adaptable robotic visual systems. This prototype, termed LGMD-UP, has the potential to reshape the implementation of bio-inspired vision in robotics. It underscores the importance of a unified framework in enhancing the flexibility and adaptability of robotics applications, facilitating the creation of more responsive and robust robotic systems capable of navigating complex environments.

In conclusion, these pioneering studies illuminate the path toward neuro-robots that deftly perceive and react to their surroundings. Inspired by the efficiency and resilience of biological systems, the researches presented here demonstrate success across diverse robotic domains. Yet, as this Research Topic underscores, navigating the complexities of neural network optimization, multi-agent collaboration, and efficient model deployment are core challenges demanding ongoing attention. These works spark optimism for the future of neuro-robotics. As more researchers explore these crucial issues, we move ever closer to creating robotic systems with adaptive intelligence that not only solve real-world problems but also contribute to our ever-expanding understanding of intelligent systems, both biological and artificial.

## Author contributions

CH: Writing—original draft, Writing—review & editing. FA: Writing—review & editing. NB: Writing—review & editing. SY: Writing—review & editing. HL: Writing—review & editing.

